# Exploring human host–microbiome interactions in health and disease—how to not get lost in translation

**DOI:** 10.1186/s13059-019-1669-4

**Published:** 2019-03-15

**Authors:** Sarah Lebeer, Irina Spacova

**Affiliations:** Department of Bioscience Engineering, University of Antwerp, Antwerp, Belgium

## Abstract

A meeting report on the 7th Wellcome Trust conference on Exploring Human Host–Microbiome Interactions in Health and Disease, held at Hinxton, UK, 5–7 December 2018.

The meeting titled *Exploring Human Host–Microbiome Interactions in Health and Disease* was held in December 2018 for the seventh time in Hinxton, Cambridge, UK. The Scientific Programme Committee once again did an outstanding job in selecting excellent keynote, invited and abstract speakers. The meeting started with a keynote lecture by John Cryan (University College Cork, Ireland) on the link between the gut microbiome, stress and brain development. The various routes of communication between the gut and brain include the vagus nerve, the immune system, tryptophan metabolism, the enteric nervous system and microbial metabolites such as short-chain fatty acids (SCFAs). These mechanisms also impinge on neuroendocrine function at multiple levels. Multifactorial modes of interaction between the microbiome and human physiological functions were a key aspect addressed at this meeting for different human body sites. This is a crucial factor to consider when translating the increased knowledge about the human microbiome into tangible human health applications. It also represents a key difference in comparison with classic approaches in medicine, where single molecules or compounds are typically selected to target single pathways in attempts to treat diseases or alleviate symptoms.

Most studies in humans are still focused on (gut) microbiome comparisons of healthy and diseased or unbalanced conditions. Such comparisons (generally by 16S amplicon sequencing) have suggested that the gut microbiota is altered in a variety of conditions such as obesity, schizophrenia, autism and Parkinson’s disease, but these relationships are—until now—mainly associations, without substantiation of real causality. Studies in animal models have the power to reveal causal relations. They have thus been key in delineating that neurodevelopment and the programming of an appropriate stress response are dependent on the microbiota. For example, Cryan presented data from an animal model that mice delivered through C-section behaved differently in acute stress situations compared with vaginal-birth controls. This opens up possibilities for studying the impact of prebiotics and probiotics and other microbiome interventions. A more extreme situation is when all microbes are eliminated, such as in germfree mice, and this model is thus increasingly being applied to study causal links between the microbiome and social behaviour. Yet, also for mice maintained in conventional nonsterile conditions, interesting causal links can be established. For instance, Cryan presented novel data showing that oral supplementation of a mixture of the three principle SCFA microbiome metabolites (acetate, propionate and butyrate) alleviated psychosocial stress-induced behaviour. In addition, SCFAs exhibited behavioral-test-specific antidepressant and anxiolytic effects, which were not present when mice had undergone psychosocial stress without impacting on the microbiome.

Yet, mice are not men. A paramount example is *Lactobacillus rhamnosus* (JB-1), which has been shown to reduce stress-related behaviour and corticosterone release and alter central expression of GABA receptors in anxious mouse models. Based on these mouse experiments, this *L. rhamnosus* strain was one of the first strains shown to be highly promising as a psychobiotic—that is, a live microorganism with a potential mental health benefit. In humans, however, this strain failed to modulate stress or cognitive performance in healthy male subjects. Thus, translating psychobiotic- and microbiota-related animal data to a healthy human population remains challenging, owing to the physiological, microbiota, immunological and social differences between humans and the stress-susceptible mouse models.

Effective translation from bench to bedside can be facilitated by a better understanding of the microbiome and probiotic and prebiotic mechanisms of action responsible for the observed health benefits. In his talk, Jack Gilbert (University of California San Diego, USA) encouraged the field to move from identifying correlations towards a more mechanistic understanding of the relationships between the human microbiome and disease through intervention trials. Recent developments in sequencing, metabolomics, proteomics and bioinformatics in combination with longitudinal sampling and multiple molecular perspectives have paved the way for microbiome-wide association studies (MWASs). Such MWASs, although highly complex in nature (Fig. 1), allow us to link the whole microbiome as a complex and dynamic system, with various aspects of health and disease, as well as treatment efficacy, and to identify targets for novel clinical interventions. This was also nicely illustrated by Paul Wilmes (University of Luxembourg, Luxembourg) in his talk on systems ecology and integrated multi-omics of human–microbiome interactions to identify key molecular transactions. However, proving causation and sufficiency remains a challenge that requires evidence from microbially mediated human intervention studies in classic diseases. A well-known example of such studies includes reports of successful treatment of *Clostridium difficile* infections through fecal microbiota transplants, for which now various follow-up trials are ongoing with more defined fecal microbial strain mixtures or formulations. More recently, Gilbert described how adjusting a patient’s diet before gut surgery could lead to a more healthy and robust microbial response to surgery-related stress factors and better surgery outcomes through the reduced presence of collagen-degrading microbes. Currently, according to ClinicalTrials.gov, more than 650 clinical trials ongoing globally involve the microbiome as a biomarker or treatment option.

**Fig. 1:**
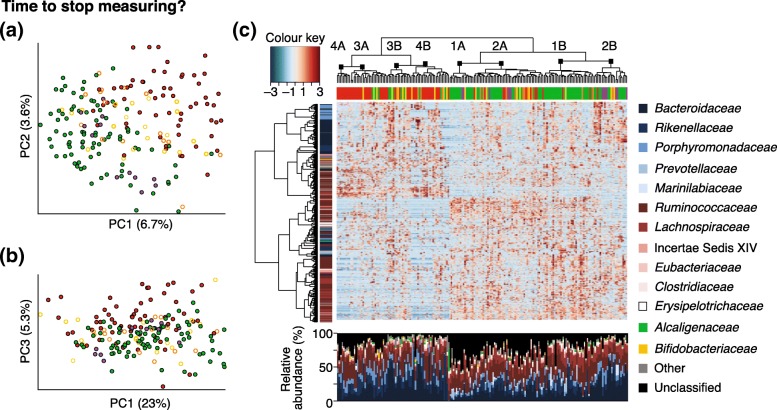
Driven by major advances in sequencing, metabolomics, proteomics and bioinformatics, an increasing number of microbiome-wide association studies (MWAS) aim to take complex and large data set analyses of the microbiome with longitudinal sampling and multiple molecular perspectives, and associate these with markers for health and disease. As discussed in the keynote debate led by Jack Gilbert and moderated by Colin Hill, it is time to cease merely measuring. It is crucial that the microbiome field moves towards more detailed functional and mechanistic studies. Various examples presented at the Wellcome Trust meeting have shown that the field is ready for the next steps in the translation of microbiome knowledge; indeed, one could assert that microbiome research is as beautiful (and as complex) as modern art. (**a**) and (**b**) show typical Principal Component Analyses (PCAs), which are often used to visualize complex, multidimensional microbiome data. (**c**) represents a typical heat map-based way of visualizing complex microbiome correlation data, with different colors representing correlation coefficients, microbiome operational taxonomic units (OTUs) and groups of subjects. More details can be found in the original paper (figure taken from Fig. 1, Claesson et al., *Nature* **488**, 178-184)

Translation of the potential of microbiome studies is also supported by the emerging evidence that microbiota members can have a profound impact on therapy efficacy and pharmacodynamics of various drugs. Laurence Zitvogel (Gustave Roussy, France) gave a convincing talk on the pathways through which the gastrointestinal microbiota and microbial factors can impact colon cancer immunotherapy and immunosurveillance. Accumulating data show that antibiotic administration can negatively alter the course of cancer immunotherapy, whereas specific microorganisms can promote positive therapy outcomes. Zitvogel described how cell-death inducers and gut microbiota members both play a role in facilitating immunogenic cell death in the ileum, which promotes immune responses against proximal colon cancer. Importantly, natural adjuvants from gut commensal microorganisms are crucial for stimulating the anticancer responses of the immune system. In particular, recent unpublished data from the Zitvogel group show that particular microbe-associated molecular patterns (MAMPs) and damage-associated molecular patterns (DAMPs) present in the ileum are highly relevant during oxaliplatin-induced death of ileal intestinal epithelial cells.

While certain microbiota can influence pharmacological treatment efficacy, there is an increasing understanding that regularly administered drugs can in turn also alter the microbiota composition. Nassos Typas (European Molecular Biology Laboratory, Germany) described a high-throughput microbiomics system that was developed as a collaborative effort of several European Molecular Biology Laboratories to screen for the interactions between representative gut bacteria and pharmacological compounds and xenobiotics. More than 1000 commercially available non-antibiotic drugs have been tested, and 24% of pharmacological compounds with human targets have been demonstrated to have an inhibitory effect on at least one of the 40 tested bacterial strains. Future research should focus on the mechanisms through which non-antibiotic drugs might promote antibiotic resistance and on determining how gut bacteria can influence the bioavailability of regularly administered drugs.

In addition to the impact of gut microbiota on exogenously administered compounds, resident microorganisms also play an important role in the metabolism of endogenous host signaling molecules. In her talk, Susan Joyce (University College Cork, Ireland) emphasized the importance of gut microbial enzymes for generating the range and variety of bile acids and salts. These bile moieties engage in local and systemic cross-talk with the host processes linked to health or disease. Consequently, metabolic (e.g. bile moieties, hormones and cytokines) and microbial markers can serve as read-outs for the health status of the host. Susan Joyce presented data from a patient cohort with inflammatory bowel diseases (IBDs), in which secondary bile acids generated by the host microbiota were linked to bile acid diarrhea and incidence of Crohn’s disease. These results demonstrate both marker correlations and a mechanistic understanding of how microbial activity can influence the host health status through alteration of host signaling molecules.

In the final part of the meeting, Julie Segre (National Human Genome Research Institute, USA) once again emphasized that humans are ecosystems constantly undergoing beneficial and potentially harmful microbe–host interactions. She used the complexity of the human skin microbiome as an example of the various multi-kingdom functional interactions that involve not only bacteria but also fungi and viruses. Interestingly, the presented longitudinal data demonstrated that skin microbial communities were specific for individuals and largely stable over months and even years of sampling. These findings are highly relevant for studies in which microbiome alterations in disease are explored and suggest that both disease development and therapeutic outcomes might be highly individualized from the microbiome perspective. In addition, Julie Segre presented data on how host genetics can define skin microbial communities, which adds another layer to the complexity of microbe–host interactions. For example, drastically increased eukaryotic viral colonization was detected in patients with the dedicator of cytokinesis-8 (DOCK8) primary immune deficiency. Hundreds of previously undescribed human papillomavirus genomes were detected in these patients through deep metagenomic sequencing, shedding light on the ‘microbial dark matter’ of the human microbiome.

In the future, sequencing and culturing data should be combined with translational microbiome approaches for a thorough characterization of how the microbiota can shape host health and disease. Detailed knowledge on the causative links between microbiota composition and functionality, and host physiology and genetics, will pave the way for personalized and precision medicine.

## Abbreviations

DAMP: Damage-associated molecular pattern
DOCK: Dedicator of cytokinesis
IBD: Inflammatory bowel disease
MAMP: Microbe-associated molecular pattern
MWAS: Microbiome-wide association study
SCFA: Short chain fatty acid

